# Effects of peroxisome proliferator activated receptor-α agonist on growth performance, blood profiles, gene expression related to liver fat metabolism in broilers fed diets containing corn naturally contaminated with mycotoxins

**DOI:** 10.3389/fvets.2022.1103185

**Published:** 2023-01-04

**Authors:** Yong Zhang, Mengchen Wang, Hao Dong, Tan Yang

**Affiliations:** College of Biological Engineering, Henan University of Technology, Zhengzhou, Henan, China

**Keywords:** broilers, liver fat metabolism, mycotoxin, performance, peroxisome proliferator activated receptor-α agonist

## Abstract

This study was conducted to determine the subclinical symptom of broilers exposure to mycotxoins from corn naturally contaminated, and the preventive effect with peroxisome proliferator activated receptor-α (PPARα) agonist (Wy-14643) supplementation. A total of 360 one-day -old male Arbor Acres broilers were randomly distributed into 4 treatments with 9 replicates of 10 birds. Dietary treatments included: treatment 1, normal corn diets group, treatment 2, normal corn + Wy-14643 diets group, treatment 3, mycotoxin-contaminated corn diets group, treatment 4, mycotoxin-contaminated corn + Wy-14643 diets group. The supplementation of Wy-14643 was added at the expense of 1 and 2 mg/kg in starter and grower diets, respectively. Birds fed mycotoxin diets had lower (*P* < 0.05) final body weight (BW), Body weight gain (BWG), feed intake (FI), and had higher (*P* < 0.05) feed conversion ratio (FCR). Feeding mycotoxin diets reduced (*P* < 0.05) the levels of serum superoxide dismutase (SOD), glutathione peroxidase (GSH-PX), catalase (CAT), total antioxidative capacity (T-AOC) and high-density lipoprotein cholesterol (HDL-C), but higher malondialdehyde (MDA), triglyceride (TG), total cholesterol (TC), low-density lipoprotein cholesterol (LDL-C), aspartate aminotransferase (AST), alanine aminotransferase (ALT) and fatty acid synthetase (FAS). The supplementation of Wy-14643 increased (*P* < 0.05) the level of serum T-AOC, but reduced (*P* < 0.05) TG and LDL-C. Interactive effect was not observed (*P* > 0.05) in growth performance and blood profiles. The relative expression of PPARα mRNA and 3-Hydroxy-3-MethylGlutaryl-CO enzyme A (HMGCoA) mRNA was higher (*P* < 0.05) in treatment 3 and treatment 4 than treatment 1 and treatment 2, and there was significant difference (*P* <0.05) between treatment 3 and treatment 4. There was significant difference (*P* < 0.05) between groups of the relative expression of recombinant carnitine palmitoyl transferase 1 (CPT1) mRNA. The relative expression of acyl CoA oxidase (ACO) mRNA was higher (*P* < 0.05) in treatment 1 and treatment 4 than treatment 2 and treatment 3, and there was significant difference (*P* < 0.05) between treatment 1 and treatment 4. The relative expression of apolipoprotein A (APO-A) mRNA was higher (*P* < 0.05) in treatment 1 and treatment 4 than treatment 2 and treatment 3. The relative expression of sterol regulatory element binding protein (SREBP) mRNA was lower (*P* < 0.05) in treatment 2, treatment 3 and treatment 4 than treatment 1, and there was significant difference (*P* < 0.05) between treatment 3 and treatment 4. Overall, feeding naturally contaminated mycotoxin diets caused negative effects on growth performance and blood profiles, while diet supplementation with Wy-14643 alleviate the detrimental effects on gene and expression related to liver fat metabolism in broilers.

## Introduction

Poultry are widely exposed to mycotoxin contaminants, which are a group of secondary metabolites or molds generated by naturally occurring metabolic processes in fungi on various cereals and feedstuffs (1). Among them, aflatoxin B_1_ (AFB_1_), ochratoxin (OA), deoxynivalenol (DON), zearalenone (ZEA), T2 toxin and fumonisin (FM) have the most toxicity to poultry health (2). It has been well-documented that various mycotoxins could result in detrimental effects on growth performance, intestinal health, immunity, hepatic function as well as biochemical and hematological parameters in broilers (1, 3). The naturally contaminated diets were more toxic than those diets with purified mycotoxins (4). In China, the corn as the major energy source accounted for over 50% of broilers feed and was extremely susceptible to mycotoxins. However, there were few literatures about the effects of corn naturally contaminated with mycotoxins of low concentrations on broilers. Previous studies demonstrated that low levels of mycotoxins may also be harmful to broilers and could alter normal metabolic functions in various organs, especially the liver (5–7).

PPARα is one of members of the peroxisome proliferator activated receptors (PPARs), which expressed in many metabolically active tissues, especially high in the liver (8). PPARα can not only differentiate adipocytes and participate in lipid and energy metabolism, but also inhibit inflammation (9).

Wy-14643 is a fibrate analog and potent agonist of PPARα. Previous studies found that the hypolipidemic effects of Wy-14643 were liver dependent and feed intake was mediated directly by PPARα activation within hepatocytes in mice (10). This may be due to the similar crosstalk between hepatic PPARα and the central nervous system in feeding behavior by fatty acid and insulin (8, 11). Wy-14643 may beneficially regulate LPS-induced inflammation in synovial fibroblasts via NF-kB pathway (12) and decrease liver injury *via* the deacetylase enzyme sirtuin1 and endoplasmic reticulum stress (13). Furthermore, Wy-14643 exert its antioxidant and anti-inflammatory effects as PPARα agonist by reducing neutrophil infiltration and proinflammatory cytokine expression and thus decrease the formation of reactive oxygen species (14–16).

The feeding of diets contaminated with mycotoxins decreases performance, alters the metabolism and induces the organs injury. It has been found that mycotoxins can be detoxified using physical, chemical, and biological methods (17, 18). Is there any other way to solve this problem? And What is the mechanism of action? PPARα might play a key role in regulating nutrient metabolism and energy homeostasis in liver. Therefore, the current experiment was conducted to determine the impact of PPARα agonist on growth performance, blood profiles, gene and expression related to liver fat metabolism in broilers fed diets containing corn naturally contaminated with mycotoxins, and to evaluate whether activating PPARα can reduce the hazard of mycotoxins to broilers.

## Materials and methods

### Experimental design and broiler husbandry

The Animal Welfare Committee of Henan University of Technology approved the animal care protocol used for these experiments (HUT20210417).

A total of 360 one-day-old male Arbor Acres broilers with an average initial body weight (BW) of 41.4 ± 0.2 g were divided into 1 of 4 dietary treatments with 9 replicates, each replicate consisting of 10 birds. The treatments were in a 2 × 2 factorial arrangement, with 2 levels of corn naturally contaminated with mycotoxins (0 and 100%) and 2 levels of Wy-14643 (1 mg/kg in the starter phase and 2 mg/kg in the growing phase). The starter phase was from d 1 to 21 and the growing phase was from d 22 to 42. All birds were housed in an environmentally controlled facility with stainless steel battery brooders. Each cage was provided with a stainless steel feeder and one nipple waterer, which allowed *ad libitum* access to feed and water throughout the experiment. All diets were fed in pellet form and formulated to meet or exceed the NRC (19) requirements for broilers (Table 1). The four treatments were fed with normal corn diets, normal corn + Wy-14643 diets, mycotoxin-contaminated corn diets and mycotoxin-contaminated corn + Wy-14643 diets, respectively. The PPARα agonist was added at the expense of corn. The temperature was kept at 33°C from 1 to 3 d of age and then it was reduced gradually to ~25°C until 14 d of age and was kept at approximately 16 to 22°C thereafter. The feed samples were analyzed for dry matter, crude protein, total ash, calcium, phosphorus and amino acids according to the standard procedures of the AOAC (20).

**Table 1:** Diet composition (as-fed basis).

**Items**	**Starter^a^**	**Grower^a^**
**Ingredients, %**		
Corn	62.00	64.00
Soybean meal	27.80	25.40
Corn gluten meal	5.00	4.00
Soybean oil	1.00	2.50
Monocalcium phosphate	1.70	1.50
Limestone	1.20	1.30
Sodium chloride	0.30	0.30
Vitamin premix^b^	0.50	0.50
Trace mineral premix^c^	0.50	0.50
**Analytical composition**		
ME, kcal/kg^d^	3,075	3,165
Crude protein, %	20.28	18.57
Dry matter, %	87.4	87.3
Total ash, %	5.02	5.00
Lysine, %	1.10	1.04
Methionine, %	0.51	0.37
Methionine + Cystine, %	0.89	0.71
Ca, %	0.97	0.86
Total phosphorus, %	0.62	0.55

a Starter diets, provided during d 1–21; grower diets, provided during d 22–42.

b Provided per kg of diet: vitamin A (from retinyl acetate), 12,000 IU; cholecalciferol, 3,000 IU; vitamin E (from DL-α-tocopheryl acetate), 12 IU; vitamin B_12_, 0.02 mg; riboflavin, 4.8 mg; nicotin amide, 18 mg; calcium pantothenate, 15 mg; folic acid, 0.5 mg; thiamine, 1.2 mg; pyridoxine, 2.0 mg; biotin, 0.25 mg; choline chloride, 1,500 mg; ethoxyquin, 80 mg.

c Provided per kg of diet: Mn (from MnSO_4_.H_2_O), 105 mg; Zn (from ZnO), 78 mg; Fe (from FeSO_4_.7H_2_O), 90 mg; Cu (from CuSO_4_.5H_2_O), 8 mg; I (from Ca (IO_3_)_2_.H_2_O), 1.8 mg; Se, 0.15 mg.

d Calculated values.

### Sampling and measurements

The content of mycotoxins in the diets was measured by ELISA method (kits, Neogen Company, MN, US; Microplate Reader, Model 680, Bio-Rad, Hercules, CA, US) according to the manufacturer's instructions.

At the end of the experiment, final BW and feed consumption each cage was determined. BWG, FI, and FCR of broilers corrected by mortality were calculated based on each cage. FCR was calculated as the ratio of feed intake to weight gain.

On d 42, two birds from each replicate were randomly selected and blood samples were collected from the jugular vein into a sterile syringe. Blood samples were then centrifuged at 3,500 x g for 15 min and serum was separated. The concentrations of SOD, T-AOC, CAT, GSH-PX, MDA, TC, HDL-C, LDL-C, and TG in the serum were analyzed using assay kits from Nanjing Jiancheng Biotechnology Institute according to the manufacturer's instructions. The activities of ALT, AST, and FAS in the serum were measured with an automatic biochemical analyzer (Model 7020, Hitachi, Tokyo, Japan) using the assay kits (Jiancheng Biotechnology Institute, Nanjing, China).

After blood collection, the same birds were electrically stunned and then sacrificed immediately by decapitation and eviscerated manually. The liver was removed manually by the same trained person. The liver samples were immediately frozen in liquid nitrogen, then stored at −80°C until gene expression analysis. The gene and expression related to liver fat metabolism were then measured. Briefly, total RNA of tissues was extracted with RNAiso Reagent (TaKaRa, Japan) using Trizol method and reverse-transcribed with RT Reagents (TaKaRa, Japan) according to manufacturer's instructions. Quantitative real-time PCR was performed using 96-well iCycler iQTM Real-Time PCR Detection System (BIO-RAD, USA). The gene-specific primers used were listed in Table 2 and purchased from TaKaRa (Japan). The PCR system consisted of 12.5 μL SYBR Green PCR Master Mix (TaKaRa, Japan), 2.0 μL of cDNA, 8.5 μL of PCR-grade water and 2.0 μL of primer pairs (100 mM forward and 100 mM reverse) for a total volume of 25 μL. The house-keeping gene β-actin was used as internal control to normalize the expression of target genes. All samples were assayed in triplicate. Cycling conditions were as follows: 95°C for 10 s, and 40 cycles involving a combination of 95°C for 5 s, 58°C for 30 s and 72°C for 30 s. The relative quantification of gene amplification by RT-PCR was performed using the value of the threshold cycle (Ct). Relative expressions of target genes were determined by the 2-ΔΔCt method.

**Table 2:** Primer sequences of genes.

**Primer**	**Primer sequences of primer**	**Accession** **number**
PPARα F	AATCGCTGGGAAATGGTC	NM_001001464.1
PPARα R	TACTCCGTAATGGTAGCCTGA	
CPT1 F	CGCCATCTGTTCTGCCTTTATG	NM_001012898.1
CPT1 R	TAACCATCATCAGCCACAGGTCC	
ACO F	ATGGTGAGGGATCAAGCCGA	NM_204188.2
ACO R	TTCTTCAGGTTGGTTTCGTGGAT	
HMGCoA F	AGGAACGCACATGGAGCA	XM_422225.6
HMGCoA R	AGTCGTCGAGGGTGAAAGG	
apo-A1 F	CATTCGGGATATGGTGGACG	NM_205525.4
apo-A1 R	CAGCCAGCTTCAGGTCAA	
SREBP F	CATTGGGTCACCGCTTCTTCGTG	NM_204126.2
SREBP R	CGTTGAGCAGCTGAAGGTACTCC	
GAPDH F	GAAAGTCGGAGTCAACGGATTT	NM_204305.1
GAPDH R	TGATAACACGCTTAGCACCA	

### Statistical analysis

All data were analyzed using the GLM procedure of SAS (38). The data were analyzed as a completely randomized design with treatments arranged in a 2 × 2 factorial, the main effects of mycotoxin-contaminated corn (0 and 100%), PPAR-α agonist (1 mg/kg in starter, 2 mg/kg in grower), and the interactions among these factors were analyzed with the least squares means using the Bonferroni test. The probability level of *P* < 0.05 was considered to be statistically significant.

## Results

### Mycotoxin content of the diets

The concentrations of AFB_1_, DON and ZEA were not detected in normal corn and normal corn + Wy-14643 diets (Table 3). The concentrations of AFB_1_, DON and ZEA were 5.6, 550.0 μg/kg, 170.7 μg/kg in contaminated corn diets, and 5.5, 550.0 μg/kg, 170.3 μg/kg in contaminated corn + Wy-14643 diets, respectively. Mycotoxins other than these were below the limits of detection.

**Table 3:** Mycotoxin content of the experimental diets.

**Items^a^**	**Normal corn**	**Normal corn + Wy-14643**	**Contaminated corn**	**Contaminated corn + Wy-14643**
AFB_1_, μg/kg	ND^b^	ND^c^	5.6	5.5
DON, μg/kg	ND^b^	ND^c^	550.0	550.0
ZEA, μg/kg	ND^b^	ND^c^	170.7	170.3

a AFB_1_, aflatoxin B_1_; DON, deoxynivalenol; ZEA, zearalenone.

b ND, not detected.

c AFB1, DON, ZEA in the diets with contaminated corn.

### Growth performance

FCR did not differ among dietary groups (*P* > 0.05). Feeding mycotoxin diets decreased (*P* < 0.05) final BW, BWG and FI (Table 4), there were no differences between contaminated corn diets and contaminated corn + Wy-14643 diets. Interactive effect was not observed (*P* > 0.05) in final BW, BWG, FI and FCR.

**Table 4:** Effects of PPAR-α agonist on growth performance in broilers fed mycotoxin contaminated corn^a^.

							***P-*value**	
**Item** ^b^	**Normal corn**	**Normal corn** + **Wy-14643**	**Contaminated corn**	**Contaminated corn** + **Wy-14643**	**SEM** ^c^	**Main effect of mycotoxin**	**Main effect of Wy-14643**	**Interactive effect of mycotoxin and Wy-14643**
Initial BW, g	41.4	41.3	41.4	41.5	0.20	0.85	0.64	0.72
Final BW, g	2,643	2,677	2,319	2,388	50.4	<0.01	0.26	0.70
**D 1-42**								
BWG, g	2,602	2,636	2,278	2,346	48.4	<0.01	0.27	0.71
FI, g	4,665	4,622	4,256	4,299	70.4	<0.01	0.89	0.68
FCR	1.79	1.75	1.87	1.83	0.02	0.02	0.17	0.85

a Means represent 9 replicates with 10 birds per cage (n = 9/treatment).

b BWG, body weight gain; FI, feed intake; F/G, feed/gain.

c Standard error of the means.

### Antioxidant activity

Birds fed with mycotoxin-contaminated diets had lower (*P* < 0.05) levels of serum SOD, GSH-Px, CAT and T-AOC, but higher (*P* < 0.05) MDA than the birds fed with normal diets (Table 5). The supplementation of Wy-14643 increased (*P* < 0.05) the level of serum T-AOC than that of mycotoxin-contaminated corn group, and dietary Wy-14643 did not influence (*P* > 0.05) serum SOD, GSH-Px, CAT or MDA. Interactions between mycotoxin and Wy-14643 were not significant (*P* > 0.05).

**Table 5:** Effects of PPAR-α agonist on antioxidant activity in broilers fed mycotoxin-contaminated corn^a^.

**Item^b^**							***P-*value**	
	**Normal corn**	**Normal corn** + **Wy-14643**	**Contaminated corn**	**Contaminated corn** + **Wy-14643**	**SEM** ^c^	**Main effect of mycotoxin**	**Main effect of Wy-14643**	**Interactive effect of mycotoxin and Wy-14643**
SOD, U/mL	224	232	196	206	4.76	<0.01	0.12	0.95
GSH-Px, U/mL	238	242	217	225	3.80	<0.01	0.28	0.70
CAT, U/mL	56	62	51	52	1.56	<0.01	0.16	0.41
MDA, nmol/mL	5.62	5.46	6.20	6.12	0.10	<0.01	0.15	0.58
T-AOC, U/mL	10.36	12.04	8.42	9.05	0.47	<0.01	0.04	0.27

a Means represent 9 replicates with 2 birds per cage (n = 18/treatment).

b SOD, superoxide dismutase; T-AOC, total antioxidative capacity; CAT, catalase; GSH-PX, glutathione peroxidase; MDA, malondialdehyde.

c Standard error of the means.

### Serum lipid

Birds fed with mycotoxin-contaminated diets had lower (*P* < 0.05) level of serum HDL-C, but higher (*P* < 0.05) TG, TC and LDL-C than the birds fed with normal diets (Table 6). The supplementation of Wy-14643 decreased (*P* < 0.05) TG and LDL-C in the serum than that of mycotoxin-contaminated corn group, and dietary Wy-14643 did not influence (*P* > 0.05) serum HDL-C or TC. No interactions were observed (*P* > 0.05) in serum lipids between mycotoxin and Wy-14643.

**Table 6:** Effects of PPAR-α agonist on serum lipid in broilers fed mycotoxin contaminated corn^a^.

**Item^b^**							***P-*value**	
	**Normal corn**	**Normal corn + Wy-14643**	**Contaminated corn**	**Contaminated corn + Wy-14643**	**SEM** ^c^	**Main effect of mycotoxin**	**Main effect of Wy-14643**	**Interactive effect of mycotoxin and Wy-14643**
TG, mmol/mL	0.41	0.37	0.61	0.55	0.03	<0.01	0.01	0.61
TC, mmol/mL	3.04	2.98	3.56	3.40	0.08	<0.01	0.29	0.63
HDL-C, mmol/mL	1.78	1.81	1.35	1.39	0.07	<0.01	0.21	0.72
LDL-C, mmol/mL	0.37	0.32	0.73	0.65	0.05	<0.01	<0.01	0.45

a Means represent 9 replicates with 2 birds per cage (n = 18/treatment).

b TC, total cholesterol; TG, triglyceride; HDL-C, high-density lipoprotein cholesterol; LDL-C, low-density lipoprotein cholesterol.

c Standard error of the means.

### Lipid metabolism-related enzyme

Birds fed with mycotoxin-contaminated diets had higher (*P* < 0.05) serum AST, ALT and FAS than the birds fed with normal diets (Table 7). Dietary Wy-14643 did not influence (*P* > 0.05) serum AST, ALT or FAS. Interactions between mycotoxin and Wy-14643 were not significant (*P* > 0.05).

**Table 7:** Effects of PPAR-α agonist on enzyme concerned with lipid metabolism in broilers fed mycotoxin contaminated corn^a^.

**Item^b^**							***P-*value**	
	**Normal corn**	**Normal corn + Wy-14643**	**Contaminated corn**	**Contaminated corn + Wy-14643**	**SEM** ^c^	**Main effect of mycotoxin**	**Main effect of Wy-14643**	**Interactive effect of mycotoxin and Wy-14643**
AST, U/L	78	69	121	110	7.09	<0.01	0.14	0.56
ALT, U/L	42	40	72	71	4.51	<0.01	0.43	0.84
FAS, U/L	289	286	344	326	8.60	<0.01	0.32	0.45

a Means represent 9 replicates with 2 birds per cage (n = 18/treatment).

b ALT, alanine aminotransferase; AST, aspartate aminotransferase; FAS, fatty acid synthetase.

c Standard error of the means.

### Gene and expression related to liver fat metabolism

**Figure 1:**
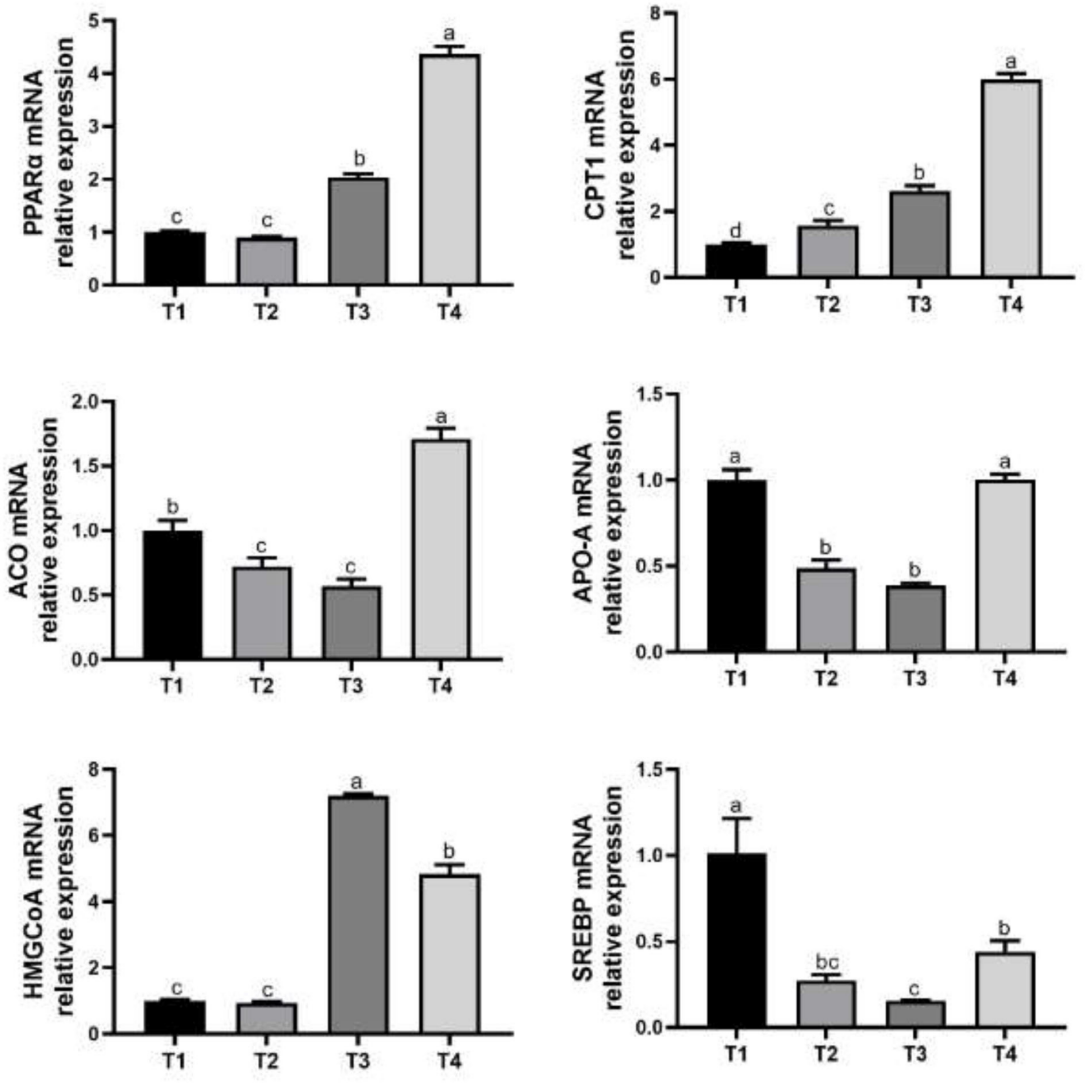
Effects of PPAR-α agonist on genes expression related to liver fat metabolism in broilers. T1, normal corn diets group; T2, normal corn + Wy-14643 diets group; T3, mycotoxin-contaminated corn diets group; T4, mycotoxin-contaminated corn + Wy-14643 diets group. The a, b, c, and d different letters indicate the significant differences.

Feeding mycotoxin-contaminated diets (treatment 1 and treatment 2) increased (*P* < 0.05) the relative expression of PPARα mRNA and HMGCoA mRNA than the birds of treatment 3 and treatment 4 (Figure 1). The relative expression of CPT1 mRNA in treatment 1 was greater (*P* < 0.05) in broilers compared with that of treatment 2, treatment 3 and treatment 4. Birds of treatment 2 and treatment 3 had lower (*P* < 0.05) relative expression of ACO mRNA, while birds of treatment 4 had higher (*P* < 0.05) relative expression of ACO mRNA compared with the birds of treatment 1. The relative expression of APO-A mRNA was lower (*P* < 0.05) in broilers of treatment 2 and treatment 3 diets compared with the birds of treatment 1. Birds of treatment 2, treatment 3 and treatment 4 had lower (*P* < 0.05) relative expression of SREBP mRNA than that of treatment 1.

## Discussion

The feeding of grain naturally contaminated with mycotoxins has been previously shown to reduce growth performance. Studies demonstrated that mycotoxins decreased FI by 9–12%, BWG by 13–14% and FCR by 7% in broilers (1, 21). Moreover, The DON (1,845 μg/kg) and ZEA (534 μg/kg) decreased BWG by 18% and increased FCR by 19% in broilers without any effect on FI (22). Others also observed the similar results in broilers fed diets containing 1,680 μg/kg DON and 145 μg/kg ZEA (23). In agreement with previous studies, feeding diets containing corn naturally contaminated low concentrations of mycotoxins decreased BWG and final BW by 12% in broilers in the current study.

Some studies have reported a modest enhancement in the body weight of chickens exposed to aflatoxins in their diet due to hormesis, which is a dose-response phenomenon characterized by low-dose stimulation (24). In the current studies, the diets were naturally contaminated with low concentrations of mycotoxins, however, no similar results were observed in growth performance, which could be explained by the fact that feeding of grains contaminated naturally with several mycotoxins sometimes resulted in toxicological synergy (25).

It is expected that the PPARα agonist (Wy-14643) may exert its lipid-lowering, antioxidant and anti-inflammatory effects on target liver, and then have the potential to improve the health status and growth performance like conjugated linoleic acid as the PPARα agonist (8). Unfortunately, no effects of the supplementation of Wy-14643 were observed on the final BW, BWG, FI or FCR. Similarly, the addition of PPARα agonist (0.5% clofibrate) also did not affect BWG, FI or FCR in broilers (26).

The co-occurrence of mycotoxins caused detrimental effects on serum antioxidant parameters in broilers in the present study, which were in agreement with previous studies. Dietary mycotoxins may result in oxidative stress, which can be characterized by increased serum MDA and decreased antioxidant enzymes activities, such as serum SOD, GSH-Px, CAT and T-AOC (27). Moreover, mycotoxins increased serum AST and ALT in broilers (3), which were consistent with our results. Similarly, the serum TC was increased in broilers fed diets containing aflatoxin B_1_, zearalenone, deoxynivalenol, and fumonisin (28), which may be attributed to the hepatotoxic effects of AFL, ZEN, FUM, and DON or their synergetic effect that characterized by impairment of transport and lipid metabolism of liver (29).

As expected, the supplementation of Wy-14643 improved the level of T-AOC in broilers fed mycotoxins diets. The data supported our inference that the increased antioxidant activity may be involved in the regulation of liver injury by Wy-14643 through PPARα in broilers fed mycotoxins diets. This was in agreement with previous studies (14–16), which indicated that Wy-14643 might prevented the formation of reactive oxygen species. A significant reduction in MDA formation in the liver of rats pretreated with Wy-14643 was previously reported (13).

PPARα deficiency led to accumulation of hepatic TG (30). PPARα reduced circulating triacylglycerol levels *via* transcriptional modulation of genes related to lipolysis, cellular uptake, β-oxidation of fatty acids, and decreased synthesis of fatty acids and TG (31). Dietary Wy-14643 exactly decreased serum TG and LDL-C in broilers fed mycotoxins diets. Previous studies demonstrated that the major physiological effects of Wy-14643 agonist acted primarily through hepatic PPARα, then activated lipid catabolism and lowered consistently TG (8, 32, 33). This study confirmed the lipid-lowering effect of Wy-14643 in broilers. Li et al. (8) proposed that PPARα activation principally facilitates lipid-lowering effects that PPARα activation is necessary for the hypolipidemic actions of PPARα agonists.

Previous studies have demonstrated that the liver gene expression involved in the immune system, antioxidant function and biotransformation can be influenced by mycotoxins in broilers (4, 22, 34). In our study, we particularly evaluated the liver gene expression on the PPARα signal pathway. PPARα is the main regulatory factor of body and liver metabolism, which can regulate liver energy consumption by up-regulating fatty acid oxidation capacity and controlling fatty acid intake (35). The mRNA expression of PPARα was increased expectedly by the mycotoxins treatments, which may reflect the liver damage characterized by the adverse effects on the serum parameters about antioxidant capacity, liver function and lipid metabolism in the present study. Our study showed that feeding mycotoxins increased the relative mRNA expression of CPT1 and HMGCoA, while decreased the relative mRNA expression of APO-A and SERBP, which may mirror the liver injury and increase in serum TC, TG, and FAS. Similarly, previous study used the perfluorooctanoic acid to create the liver injury model and observed that the level of PPARα mRNA was increased, while the ACO and APO-A mRNA expression was decreased in pregnant female rats (36).

PPARα has been previously shown to upregulate many genes involved in fatty acid utilization, including several genes for hepatic clearance of LDL-C, fatty acid uptake, fatty acid activation and transport into the mitochondria, peroxisomal and mitochondrial β-oxidation, and some enzymes for mitochondrial respiration (37). The agonist of PPARα (Wy-14643) has been proven to increase the mRNA relative expression of PPARα, CPT1, ACO and HMGCoA, while decrease relative expression of SREBP mRNA in broilers fed mycotoxins diets. This indicated that the agonist of PPARα (Wy-14643) exactly activated PPARα and interfered the lipid metabolism.

However, to the best of our knowledge, this is the first study to evaluate the PPARα agonist (Wy-14643) in broilers fed diets containing corn naturally contaminated with mycotoxins. More studies are needed to determine the effects of Wy-14643 on broilers.

## Conclusion

This study indicates that diets contaminated with mycotoxins could reduce growth performance and antioxidant capacity. Although the supplementation of Wy-14643 in mycotoxin-contaminated diets failed to mitigate the adverse effects on growth performance, the addition of Wy-14643 could partially enhance the antioxidant capacity and lipid-lowering effects in broilers.

## Data availability statement

The original contributions presented in the study are included in the article/supplementary material, further inquiries can be directed to the corresponding author.

## Ethics statement

The animal study was reviewed and approved by the Animal Welfare Committee of Henan University of Technology approved the animal care protocol used for these experiments (HUT20210417). Written informed consent was obtained from the owners for the participation of their animals in this study.

## Author contributions

YZ: writing-reviewing and editing and funding acquisition. MW and HD: methodology. MW: writing—original draft HD: data curation. HD and TY: formal analysis. All authors contributed to the article and approved the submitted version.
